# Exploring patient experiences of participating in a real and sham dry cupping intervention for nonspecific low back pain: A qualitative study

**DOI:** 10.1371/journal.pone.0268656

**Published:** 2022-05-19

**Authors:** Hugo Jário Almeida Silva, Mariana Arias Avila, Kamilla Maria Sousa Castro, Yago Tavares Pinheiro, Caio Alano Almeida Lins, Germanna Medeiros Barbosa, Marcelo Cardoso de Souza

**Affiliations:** 1 Faculdade de Ciências da Saúde do Trairi, Postgraduate Program in Rehabilitation Sciences, Universidade Federal do Rio Grande do Norte, Santa Cruz, RN, Brazil; 2 Physical Therapy Department, Universidade Federal de São Carlos, São Carlos, São Paulo, SP, Brazil; Mugla Sitki Kocman Universitesi, TURKEY

## Abstract

**Background:**

The current quality of evidence supporting dry cupping for individuals with chronic low back pain (CLBP) is low and suggests that nonspecific factors impact experiences reported by patients. Therefore, this study assessed the impacts of social and professional support on the experience of individuals with CLBP treated with dry cupping or sham.

**Method:**

This is an observational study with qualitative approach. Twenty-four individuals with CLBP who received dry cupping or sham in a previous clinical trial were invited. Data was collected using a semi-structured interview conducted by a trained researcher. Content analysis was used to analyze experiences, systematic procedures, and description of the content of messages. The dimensions of “pain”, “general perceptions”, and “perceived social and professional support” guided the analysis.

**Results:**

Answers of both groups converged on similar perceptions, especially regarding pain. Physical condition was the most fragile aspect. We also observed an influence of perceived social and professional support on painful symptoms. Thus, the experience of individuals with CLBP treated with dry cupping or sham indicated that factors related to social and professional support impacted results.

**Conclusions:**

We observed that individuals with CLBP reported similar perceptions of the effects of dry cupping or sham treatment, indicating that contextual factors may influence the perception of these individuals regarding the treatment received.

## Introduction

Chronic low back pain (CLBP) is a public health problem in several countries associated with high levels of pain and disability [1–3]. This condition is highly prevalent in males and females and negatively impacts quality of life [4]. In this sense, a multi-professional approach (including physiotherapists) is recommended for individuals with this condition [5].

Among therapeutic possibilities, dry cupping has been used [6, 7] to reduce pain [8, 9], improve physical function [8, 10], and promote better quality of life of individuals with CLBP [11, 12].However, the current quality of evidence supporting the technique is low [10], and its effects may not be superior to sham [7, 13]. Therefore, improvements observed in individuals with CLBP after dry cupping may be due to nonspecific factors, such as therapeutic alliance. This notion is defined as the positive connection between therapist and patient, generated by a collaborative relationship based on professional support, empathy, and mutual respect [14], which may improve therapeutic intervention results and influence psychological and general health status, physical function, and perception of patients to the treatment [15–17].

Although specific interventions may not fully explain therapeutic results [18], the influence of nonspecific factors (including therapeutic alliance) on therapeutic results of individuals with CLBP must be assessed [19]. In this sense, comprehending all components responsible for clinical outcome alterations may help understand therapeutic changes, improve professional practice, and develop more effective approaches [20].

Therefore, this study aims to analyze the perceived experience of individuals with CLBP submitted to real dry cupping treatment or sham and its association with the dimensions of pain, physical condition, and perceived social and professional support.

## Materials and methods

### Design

This exploratory and descriptive study with a qualitative approach explored opinions and representations on the topic investigated. The content analysis proposed by Bardin [21] was used to analyze experiences, systematic procedures, and objectives for describing the content of messages. In this study, content analysis was structured in thematic categories, categorized, and grouped.

The Consolidated Criteria for Reporting Qualitative Research was used to ensure a complete and transparent reporting of this study [22]. All participants provided written informed consent, and the research ethics committee of the Federal University of Rio Grande do Norte, Faculty of Health Sciences of Trairi (FACISA/UFRN) approved the study (number:XXXXXXX).

### Participants, recruitment, and sample size

Between February and July 2020, participants from an ongoing randomized controlled trial (RCT) study [23] (ClinicalTrials.gov–NTC03909672) who completed a 2-month intervention were consecutively invited to participate in this qualitative study. People were eligible for inclusion if presented low back pain for ≥ 3 months, pain intensity between 3 and 8 on a numerical pain rating scale, age between 18 and 59 years, and body mass index of < 35 kg/m^2^. Exclusion criteria were: individuals who had ever been treated with dry cupping or were undergoing physiotherapy; presence of any contraindication for dry cupping therapy; presence of neurological, vestibular, visual, or auditory deficits that could interfere with assessments; signs of fractures, inflammatory diseases, infection, or tumors in the spine; radiating lumbar or sacroiliac pain; rheumatic diseases (e.g., fibromyalgia or ankylosing spondylitis); travel plans in the next two months; and those unable to properly complete the assessment for any reason.

Sample size was calculated based on saturation of responses obtained with participants since new interviews would add few elements to the discussion. Nevertheless, sample size could be defined according to the experience of researchers and theoretical understanding of what is proposed in the study [24]. From 90 participants (45 per group) enrolled in the RCT, a maximum of 33.3% (n = 30) was estimated to compose the sample. However, 24 patients agreed to participate in the study.

### Data collection

A semi-structured interview was performed between February and July 2020 (six months after the and off the intervention) to collect data regarding the experiences of individuals with CLBP submitted to dry cupping or sham. Data obtained were evaluated according to Bardin [17] and Minayo [20].

### Pre-interview interventions

**Fig 1:**
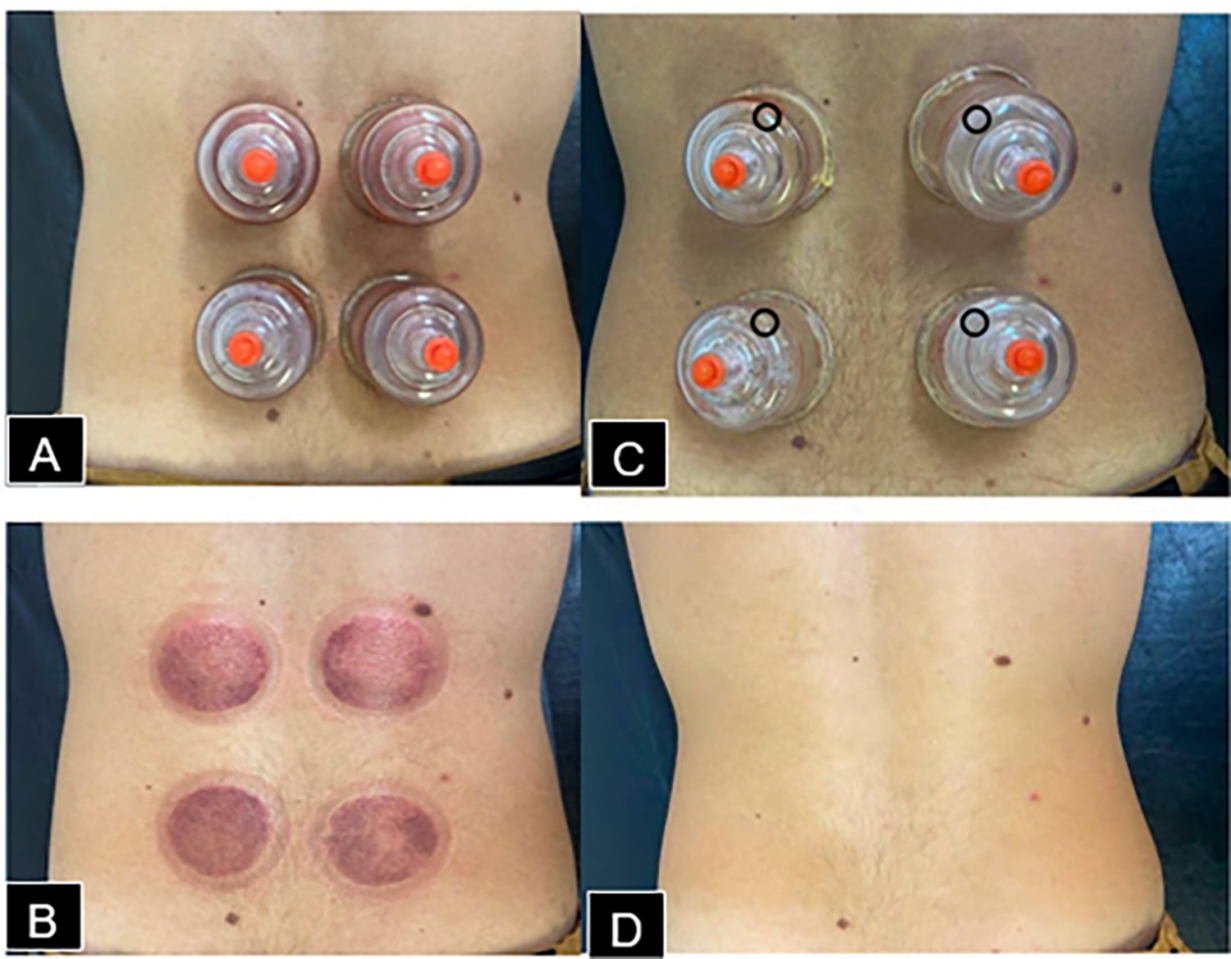
Real Dry cupping sites during (A), after (B) intervention; Sham Dry cupping Sham during (C) and after (D) intervention. The black circles(C) demonstrate the sites with holes.

The intervention protocol was published in detail elsewhere [25]. Initially, participants were informed about objectives and procedures of the study, followed by the intervention (dry cupping or sham). An experienced physiotherapist applied interventions individually in a quiet university outpatient clinic, with the participant positioned prone and relaxed [25]. Dry cupping therapy was applied using two acrylic size 1 cups (4.5 cm internal diameter) with a distance of 3 cm between each cup, bilaterally, and parallel to L1–L5 vertebrae. The real dry cupping group performed two suctions for 10 min, once per week, for eight weeks. Sham group received the same protocol; however, cups were prepared to release the negative pressure in a few seconds [25]. During the study, interactions between therapists and patients occurred only during application of the technique (Fig 1).

### Post-intervention interview

Interviews were performed to obtain information regarding experiences and understand the perception of individuals regarding the technique (dry cupping or sham) six months after the protocol. All interviews were conducted by an experienced physiotherapist not involved with other study procedures. She was a graduate student and research volunteer at the time of the study.

Data were collected by telephone, and participants were also allowed to write impressions, photograph, and send to researchers using messaging applications. Technological resources captured responses in full and provided information to create a qualitative database. Pseudonyms were assigned to participants for confidential purposes, and all answers were stored in folders and individually analyzed. Participants were allowed to report perceptions, without time or character limitation, while responding to the following questions: 1) “Tell us a little about the experience with dry cupping treatment”; 2) “How do you perceive social and professional support during the treatment?”. The way of directing questions encouraged participants to freely express perceptions regarding the treatment in the dimensions of pain, general perceptions, and perceived social and professional support. These dimensions were defined based on reports and complaints of participants during the intervention and reflections consulted previously [26–28].

### Data analysis

Reports were transcribed and reviewed according to consistency of established questions. In the end, another researcher (KMSC) described and categorized opinions into the following dimensions of treatment effects: pain, general perceptions, and perceived social and professional support. Subsequently, we identified dimensions corresponding to perceptions, highlighted in the text, and grouped using an analytical framework. As suggested by Minayo [20], statements were carefully interpreted, and investigated contexts were considered to decompose data and identify relationships between perceived experience and dimensions.

## Results

Twenty-four individuals (16 female and 8 male, mean age of 23 ± 6.3 years) were included; eleven individuals completed high school. As shown in Table 1, twelve individuals composed each group.

**Table 1:** Characteristics of participants included in the real dry cupping (RG = 12) and sham (SG = 12) groups.

Pseudonym	Age (years)	Gender	Educational level
Real dry cupping group			
GR1	27	Male	Incomplete higher education
GR2	18	Female	Incomplete high school
GR3	41	Female	Complete high school
GR4	23	Male	Complete high school
GR5	35	Male	Complete higher education
GR6	20	Female	Incomplete higher education
GR7	20	Male	Complete high school
GR8	28	Male	Incomplete higher education
GR9	18	Female	Complete high school
GR10	19	Male	Complete high school
GR11	23	Male	Complete high school
GR12	22	Female	Incomplete higher education
Sham group			
SG1	25	Female	Complete higher education
SG2	18	Female	Complete high school
SG3	19	Female	Incomplete high school
SG4	19	Female	Incomplete higher education
SG5	23	Female	Incomplete higher education
SG6	37	Male	Complete Higher Education
SG7	19	Female	Incomplete higher education
SG8	24	Female	Incomplete higher education
SG9	21	Female	Complete high school
SG10	30	Female	Complete high school
SG11	18	Female	Complete high school
SG12	22	Female	Complete high school

RG = real dry cupping group, SG = sham group.

Categorization, grouping, interpretation, and data presentation using a qualitative approach were valuable for this study since the perceived effects varied between participants. Thus, the design of treatment dimensions was related to descriptions during treatment (Box 1).

**Box 1. Summary of established dimensions and post-intervention effects**

**Table:** 

Dimensions	Description of effects
Pain	This variable includes individual, sensory, or emotional experiences causing discomfort or relief expressions.
General perceptions	This variable involves function and conditions of individuals for other activities of daily living, relaxation, beliefs, sleep, and other perceptions.
Perceived social and professional support	This variable is related to perception and satisfaction with professional assistance, reception, and support from family, friends, and/or the community, and importance given by third parties to factors generating individual discomfort.

As shown in Boxes 2 and 3, results regarding treatment perception were analyzed following the order of data description, analysis, and interpretation. Participants from real dry cupping group were assigned with the acronym RG followed by a number, whereas perceptions of participants from sham group were identified using the acronym SG followed by a number.

**Box 2. Descriptive results of perception of participants related to guiding questions according to the dimensions of pain and general perceptions**

**Table:** 

DIMENSIONS	PERCEPTION
**Pain**	***RG1 “****My* ***experience*** *with the dry cupping was very* ***innovative****; I had never undergone any procedure of this nature*.*”* ***SG1 “I did not feel any changes*** *in my body after the dry cupping treatment*, *and everything remained the same*.*”*
	***RG2*** *“In general*, *the* ***experience*** *was very* ***satisfactory***, *I was well informed about the purpose of the project and the procedures before I even agreed to participate*, *[*.* *.* *.*] the* ***pain decreased*** *during the treatment process*.*”* ***SG2 “****The treatment with the dry cupping was*, *in summary*, ***very profitable***. *The responsible professionals offered all the necessary assistance*, *I felt differences in my posture*, *and the* ***low back pain was ameliorated*.***”*
	***RG3*** *“To be honest*, ***I did not perceive any results*** *[*.* *.* *.*]*.*”* ***SG3 “****For my experience*, *I have to say that* ***I created some expectations***, *which* ***were not met*** *in the end [*.* *.* *.*]*.*”*
	***RG4 “****It was a sensational experience*. *[*.* *.* *.*] I liked the result*. *Nowadays*, ***I don’t feel pain anymore*.***”* ***SG4 “****The dry cupping project was* ***very interesting*** *because it showed me the countless possibilities for the treatment of people with low back pain*, *it was also important to* ***relax*** *because*, *during the application of the dry cupping*, *the patient was more relaxed*, *and this always caused a* ***good feeling*** *[*.* *.* *.*]*.*”*
	***RG5 “****[*.* *.* *.*] The sensation that the application caused after each session was good*, *pleasant; I* ***felt relief in my lower back pain***. *I wish there were more sessions*.*”* ***SG5 “****[*.* *.* *.*] I continued the treatment betting on the results at the end because*, *even though I had the possibility of a placebo*, ***I felt better*** *after the dry cupping*, ***the pain was minimized*** *[*.* *.* *.*]*.*”*
	***RG6 “****The* ***experience*** *was* ***very satisfactory*** *because*, *in addition to being introduced to a new technique*, *I was able to understand more about its benefits*. *During the therapy*, *I* ***did not feel any improvement***, *but today I realize that the* ***pain exists but is not compared to the one before the treatment*.***”* ***SG6 “****My experience with the dry cupping treatment* ***was something new*** *[*.* *.* *.*]*. *I have perceived some results from the first session because I used to fell* ***a lot of pain*** *in my lower back*. ***After eight sessions*** *[*.* *.* *.*]*, *I was* ***much better from the pain*.***”*
	***RG7 “****The dry cupping treatment was a* ***new experience*** *for me*, *in which I consulted once a week*, *to relieve and* ***improve some pain and discomfort*** *in my lower back*. *As the days went by*, *tests were performed to notice some kind of evolution*.*”* ***SG7 “****My* ***experience*** *with the dry cupping treatment* ***was great*.** *Until then I didn’t know it*.*”*
	***RG8 “****I only* ***felt improvements during*** *the treatment*, *but* ***after the end*** *of the treatment*, ***the pain returned*.***”* ***SG8 “****I emphasize that*, *during the meetings for the application of the dry cupping in the* ***lower back***, *I always* ***felt comfortable***, *and nothing would make me want to interrupt the treatment*. *After the application*, *I felt* ***some relief*** *in my* ***low back pain*** *[*.* *.* *.*]*.*”*
	***RG9*** *“It was a* ***very good and surprising experience***, *better than I thought*.*”* ***SG9 “****It was an experience that made me realize that my back pain could become something more serious*. *With the dry cupping treatment*, *I noticed that by the time that the end of the treatment was coming*, *I* ***felt some relief for a certain time*.***”*
	***RG10 “****The dry cupping had every way to heal*, *relieve everything*, *etc*. *However*, *it was* ***very normal***, *the sessions were good*, *it was* ***good to feel that pressure*** *on my lower back*, *but it lasted for a short time [*.* *.* *.*]*.*”* ***SG10*** *“I* ***felt relaxed***, *I wanted to sleep*, *and the best thing was that the skin did not turn purple in the region where the treatment was applied*, *the treatment with the dry cupping was very good*, *it* ***improved my lower back pain*.***”*
	***RG11 “****[*.* *.* *.*] as the days went by*, *and the sessions progressed*, *I* ***noticed the best improvement*** *of the low back pain [*.* *.* *.*]*.*”* ***SG11 “****My* ***experience*** *with the dry cupping was* ***wonderful***, *during the application* ***I didn’t feel any significant pain***, *it didn’t turn purple for several days*, *and the* ***pain left*** *after the application [*.* *.* *.*]*.*”*
	***RG12 “****My* ***experience with the treatment was wonderful***, *I thought it would be painful*, *but it was the other way around*. *[*.* *.* *.*]* ***and the low back pain has reduced*.***”* ***SG12 “****My experience with the dry cupping was to* ***obtain a satisfactory improvement [*.* *.* *.*]*.*”***
General perceptions	***RG1 “****The first sessions were a little bit strange due to the* ***purple marks***, *it is a little* ***difficult to get used*** *to them since they were located on the back*, *I kind of forgot that they existed and sometimes scared me when I noticed them [*.* *.* *.*]*.*”* ***SG1 ****(Information not obtained)*
	***RG2 “****[*.* *.* *.*] regarding the physical state*, *the* ***result*** *was also* ***positive*** *[*.* *.* *.*]*.*”* ***SG2 “It reduced the difficulty of stretching*** *and performing other* ***physical exercises***, *as well as* ***improved sleep quality*.***”*
	***RG3 “****I kept going to perceive if there would be any* ***progress***, *but* ***it didn’t***. *Unfortunately*.*”* ***SG3*** *(Information not obtained)*
	***RG4 “****[*.* *.* *.*] At the beginning of everything*, ***I did not believe*** *that this* ***treatment would work***, *but the days went by*, *and I was adapting to the dry cupping treatment*.*”* ***SG4*** *(Information not obtained)*
	***RG5 “****[*.* *.* *.*] my experience with the dry cupping treatment is that I felt the* ***effects on my body***.*”* ***SG5 “****[*.* *.* *.*] the treatment became the time of day that I could* ***relax from all the stress of the college routine*** *and* ***receive care*** *from the team*.*”*
	***RG6*** *(No physical impacts were reported)* ***SG6*** *(Information not obtained)*
	***RG7*** *(No physical impacts were reported)* ***SG7 “My change was remarkable*** *every week because I felt* ***more and more relaxed*** *right after each session*.*”*
	***RG8*** *(No physical impacts were reported)* ***SG8*** *(Information not obtained)*
	***RG9*** *(No physical impacts were reported)* ***SG9*** *(Information not obtained)*
	***RG10 “****[*.* *.* *.*] soon after and throughout the treatment*, ***it was as if nothing had happened***.*”* ***SG10*** *(Information not obtained)*
	***RG11*** *“[*.* *.* *.*]* ***relief from daily exhaustion***. *So it was profitable and very positive for me [*.* *.* *.*]*.*”* ***SG11*** *“[*.* *.* *.*] I think the* ***treatment was wonderful*.*”***
	***RG12*** *“[*.* *.* *.*]* ***I felt very relaxed*** *and* ***lighter during all sessions***.*”* ***SG12*** *“satisfactory improvement*, *[*.* *.* *.*] during the* ***treatment period*.***”*

RG = real dry cupping group, SG = sham group.

**Box 3. Descriptive results of perception of participants related to the dimension of perceived social and professional support**

**Table:** 

**Perceived support: social and professional**	***RG 1 “****[*.* *.* *.*] an innovative treatment that serves not only as a complementary treatment for some bodily symptoms but also aims to strengthen the bond between professionals and the Basic Health Unit users*, *for example*.*”* ***SG1*** *(Information not obtained)*
	***RG 2 “****[*.* *.* *.*] the* ***receptivity*** *of the participants and professionals of the project contributed to a better experience*. *Social support started from the moment the* ***“service”*** *was offered free of charge to the population*, *benefiting not only the community but also the professionals and students involved in the project*, *whose aim was to* ***improve the already studied practices*.***”* ***SG2*** *“The* ***professional support became the differential*** *in the dry cupping treatment*, *helped in the physical aspect*, *enabled better performance of the process and ended up [*.* *.* *.*]* ***assisting psychological issues*** *since it was a moment of relaxation and distraction*.*”*
	***RG 3*** *“Well*, *the* ***social and professional support*** *issue was very rewarding for me because*, *in all the appointments*, *I was always very* ***well received*,** *and I felt* ***very comfortable*** *with everyone who attended me*.*”* ***SG3 “I didn’t have much social support*** *for being an* ***introvert person*** *and not sharing much of my life with other people*. *Regarding the* ***professional aspect***, *the personnel were* ***always very helpful*** *and provided total security and necessary guidance”*.
	***RG4**** “At the beginning*, *when I initiated the treatment***, *my mother saw those purple marks*** *when I got home and didn’t understand*. *I sat down and explained the purpose of the treatment to her and when she understood*, ***she supported me***. ***My colleagues supported me too***.*”* ***SG4*** *“****The social and professional support was of paramount importance during the treatment*** *to build a better relationship with patients*, *and because of this relationship*, *the* ***treatment became pleasurable***, *and contributed more to the well-being*.*”*
	***RG 5*** *“[*.* *.* *.*] I perceived the* ***social and professional support*** *of the treatment based on how the* ***project involved many people***, *with different stories and common objectives*: ***to seek an answer to their concerns*.***”* ***SG5*** *“The* ***support received*** *by the project members was* ***full of care*,** *and they were very attentive*. *Attention was paid to the environment as I felt more comfortable with the light*, *the sound*, *the temperature of the place*, *leaving the environment very cozy*. *[*.* *.* *.*] I had many benefits from the experience*, *especially related to the* ***improvements on pain*, *postural awareness*, *mental health*, *and habits changes*** *that were contributing to aggravating the pain*.*”*
	***RG6**** “Social support is presented* ***through the care and attention*** *that* ***physiotherapists*** *showed when explaining*, *informing*, *and caring for each patient who was part of the research*. *My experience was very satisfactory both for the treatment and professionals*.*”* ***SG6 “****[*.* *.* *.*] Due to the fact that* ***it is something new for the society*** *in general*, *it should be more advertised*. *This type of treatment is most seen in the world of sports*. *[*.* *.* *.*] I was* ***very well guided by the physiotherapists*** *who conducted the treatment*.*”*
	***RG 7*** *“The importance of the project to the city is* ***notorious***, *mainly due to both the* ***professionals’*** *supervision and development*, *many citizens will have a place for treatment and recovery*, *thus improving their quality of life*.*”* ***SG7*** *“I realize that it was of* ***great importance*** *since it helped to improve our* ***health***, *not only* ***physical*** *but also* ***psychological*.***”*
	***RG8*** *“Qualified professionals*, ***excellent service***, *but I* ***only felt the effects during the treatment*.***”* ***SG8 “****[*.* *.* *.*] Everyone treated me with an* ***extremely humanized*** *and* ***ethical way***, *which* ***helped me to relax*** *from the beginning to the end of the treatment*. *[*.* *.* *.*] Therefore*, *a* ***horizontal relationship*** *between professional and patients was established*.*”*
	***RG9*** *“The* ***support*** *during the treatment* ***was very nice***, *mainly regarding the explanation of the treatment and doubts*.*”* ***SG9*** *“****Social support was essential*** *so that you* ***didn’t give up on your treatment***, *and they were always encouraging*. *The* ***professional*** *support was of* ***paramount importance*** *from the good morning that was given*, *until the goodbye when it ended [*.* *.* *.*]”*
	***RG10*** *“****The social support was even funny***, *people saw the purple marks and asked what it was like*, *if it was good*, *if it really worked*, *as if it were something very famous*. *The* ***professional was always very neutral***, *neither supporting nor going the opposite way*, *making it clear that it could work or not*.*”* ***SG10*** *“****The family support was essential*** *for my treatment*, *and they always* ***helped me*.** *Regarding the* ***professional aspect***, *the physiotherapist was an excellent educator and* ***very attentive to me***.*”*
	***RG11*** *“****The social support in my experience was positive*** *concerning friends and family; some were curious about how it worked and also wanted to learn about the dry cupping treatment*. *The* ***professional part*** *was also quite* ***remarkable*** *[*.* *.* *.*]”*. ***SG11*** *“The* ***social support*** *was essential*, *especially* ***family support***. *In addition*, *direct contact with the* ***professional***, ***reception***, *and treatment continuation*.*”*
	***RG12*** “*Regarding the social and professional support*, *I perceived that* ***I was heard during the sessions***, *with great attention*, *ethics*, *and care”*. ***SG12*** *“The* ***social factor is undeniable*** *by many*, *and it is* ***important in any treatment***. *Regarding the* ***professionals*,** *they performed their function with* ***dedication*** *and were totally* ***patient***, *thus facilitating the* ***exchange of information*** *and improving the performance of the activities*.*”*

RG = real dry cupping group, SG = sham group.

### Pain and general perceptions

Perceptions of participants from both groups were similar, especially regarding pain since it is a notorious context easily perceived through positive or negative impacts. In this sense, expressions were intentionally directed to the dimension of pain, while the dimension of general perceptions was ignored or intrinsic to pain. It is worth noting that the real dry cupping group (RG) indicated pain relief during the procedure, and the return of symptoms was less intense. Complaints regarding unsuccessful results were also reported, but to a lesser extent, in the face of expectations for treatment. Although other participants received a sham approach, the reported experience and benefits in the dimension of pain were satisfactory and recommendable to other people. As expected, some participants did not report any symptom change.

Therefore, general perceptions and perceived support indicated that physical condition was the weakest aspect in this study since dry cupping presented visible physical repercussions (i.e., purple marks) that could result in dissatisfaction. However, improvements inherent to general perceptions (i.e., flexibility, sleep quality, relaxation, and relief from daily exhaustion) also deserve attention.

### Perceived support: Social and professional

Regarding the dimension of perceived social and professional support, the influence of support received by people involved in the personal or professional context on painful symptoms was substantial. The considerable involvement of participants of both groups with this dimension can also be highlighted, such as “*family support as a determining factor for treatment continuation*”, “*friends support*”, “*social stratification regarding the technique used*”, “*horizontal therapist-patient relationship*”, “*reception*, *ethics*, *and clarifications*”, and “*listening*, *dialogue*, *and encouragement*”. Participants considered these aspects as fundamental elements in the care process, with positive impacts on physical, well-being, and emotional and psychological condition.

## Discussion

To our knowledge, this is the first qualitative study aiming to understand the influence of social and professional support on experiences of individuals with CLBP treated with dry cupping. All individuals reported positive and similar perceptions in all dimensions after treatment, regardless of the technique used (real or sham).

The perception of pain improvement reported by most individuals of both groups can be explained by the expectation created before treatment [29] and other nonspecific factors, such as therapist-patient relationship [30], regression to the mean [31], and placebo effect [32]. To date, only one quantitative study observed significant improvements in pain after dry cupping compared with sham [8]. However, the study presented several methodological limitations (e.g., flaws in the randomization process and small sample size) that led to a questionable conclusion. In this sense, we conducted a clinical trial with good methodological quality to reduce bias observed in previous studies [23], in which we observed that the application of real or sham dry cupping showed no difference in the clinical improvement of patients with CLBP.

A recent systematic review also suggested that dry cupping reduced pain in patients with CLBP [7]; however, only two studies with high heterogeneity and without a comparative sham group were included in the review. Conversely, a small number of individuals receiving dry cupping therapy reported negative experiences in our study, probably due to the expectation created before treatment. This negative interpretation may be present in patients with pain since it is an individual experience influenced by other external factors [27, 33].

Regarding physical condition, many participants reported no positive effects, which led us to believe that dry cupping did not favor this outcome. Nevertheless, it is worth noting that this aspect is strongly influenced by psychological, social, and physical factors [34, 35]. A quantitative study observed that physical condition improved significantly after dry cupping compared with sham; however, the study also presented methodological limitations, such as inadequate randomization and lack of sample size calculation, blinding, and intention to treat analysis [7]. These biases may overestimate effects and generate doubtful results.

Individuals with CLBP, regardless of the technique received, reported satisfactory experiences about the received social and professional support. This may explain the positive results observed in the dimensions of pain and general perceptions since individuals receiving social support may present better results [36, 37]. Reports regarding the presence of bruise or lack of previous experience with the technique corroborate with the study of Rossetini et al. [38], who stated that “new” or “innovative” therapies administered on the skin and with high marketing characteristics presented a great placebo effect. Therefore, the positive experience reported by individuals with CLBP treated with dry cupping or sham led us to understand the mechanisms of the technique and how participants perceived this “new” treatment. We also identified that the association between dry cupping and social and professional supports are essential for therapeutic success; thus, encouraging treatment continuation and, consequently, symptom relief.

The therapist-patient alliance can also be emphasized in our study. Both groups positively reported the presence of the physiotherapist in the symptom improvement process. Literature shows how this alliance benefits patients with CLBP [14]. It is worth mentioning that the treatment was individualized and performed in a temperature-controlled and cozy room [25]. As a result, attention was entirely focused on the individual, a factor that may generate positive effects.

This study presents limitations. We did not develop a focus group to understand the perceptions of collective efficacy or accurately identify divergences and convergences between therapeutic processes and treatment effects on CLBP symptoms. Despite this, our results can assist clinicians with CLBP management by understanding factors unrelated to the technique that also impact treatment success. Last, the presence of memory bias cannot be ruled out since the study was conducted six months after the clinical trial. Nevertheless, participants felt free to report experiences.

Our findings demonstrated that individuals with chronic low back pain presented similar perceptions of the effects of dry cupping and sham treatment. Therefore, improvements reported indicate that contextual factors, such as social and professional supports, potentiate the experience of participants.

## Supporting information

S1 File (XLSX)
